# Supplementary material for: Instability of Short Arm of Acrocentric Chromosomes: Lesson from Non-Acrocentric Satellited Chromosomes. Report of 24 Unrelated Cases
Source: Int J Mol Sci. 2020 May 13;21(10):3431. doi: 10.3390/ijms21103431 (PMC7279238; doi:10.3390/ijms21103431)
Supplement: Supplementary file 1 [file ijms-21-03431-s001.pdf]

International Journal of
*Molecular Sciences*

MDPI

*Article*

# Instability of Short Arm of Acrocentric Chromosomes: Lesson from Non-Acrocentric Satellited Chromosomes. Report of 24 Unrelated Cases

**Serena Redaelli [1,§], Donatella Conconi [1,§], Nicoletta Villa [2], Elena Sala [2], Francesca Crosti [2], Cecilia Corti [3], Ilaria Catusi [3], Maria Garzo [3], Lorenza Romitti [4], Emanuela Martinoli [5], Antonella Patrizi [6], Roberta Malgara [6], Maria Paola Recalcati [3], Leda Dalprà [1,2], Marialuisa Lavitrano [1], Paola Riva [5], Gaia Roversi [1] and Angela Bentivegna [1,*]**

1. School of Medicine and Surgery, University of Milano-Bicocca, 20900 Monza, Italy; serena.redaelli@unimib.it (S.R.), donatella.conconi@unimib.it (D.C.), leda.dalpra@unimib.it (L.D.), marialuisa.lavitrano@unimib.it (M.L.), gaia.roversi@unimib.it (G.R.)
2. Medical Genetics Laboratory, Clinical Pathology Department, S. Gerardo Hospital, 20900 Monza, Italy; n.villa@asst-monza.it (N.V.), elena.sala@asst-monza.it (E.S.), f.crosti@asst-monza.it (F.C.)
3. Medical Cytogenetics Laboratory, Istituto Auxologico Italiano IRCCS, 20095 Cusano Milanino, Italy; ceciliacorti@alice.it (C.C.), i.catusi@auxologico.it (I.C.), m.garzo@auxologico.it (M.G.), p.recalcati@auxologico.it (M.P.R.)
4. Pathology and Cytogenetics Laboratory, Clinical Pathology Department, Niguarda Ca' Granda Hospital, 20162 Milan, Italy; lorenza.romitti@ospedaleniguarda.it
5. Medical Genetics Laboratory, Medical Biotechnology and Translational Medicine Department, University of Milan, 20090 Milan, Italy; emanuela.martinoli@unimi.it (E.M.), paola.riva@unimi.it (P.R.)
6. Medical Cytogenetics Laboratory, Clinical Pathology Department, San Paolo Hospital, 20142 Milan, Italy; antonella.patrizi@asst-santipaolocarlo.it (A.P.), roberta.malgara@asst-santipaolocarlo.it (R.M.)

* Correspondence: angela.bentivegna@unimib.it; Tel.: +39-0264488133

§ These authors share co-first authorship.

Received: 1 April 2020; Accepted: 11 May 2020; Published: date

**Abstract:** Satellited non-acrocentric autosomal chromosomes (ps–qs-chromosomes) are the result of an interchange between sub- or telomeric regions of autosomes and the p arm of acrocentrics. The sequence homology at the rearrangement breakpoints appears to be, among others, the most frequent mechanism generating these variant chromosomes. The unbalanced carriers of this type of translocation may or may not display phenotypic abnormalities. With the aim to understand the causative mechanism, we revised all the ps–qs-chromosomes identified in five medical genetics laboratories, which used the same procedures for karyotype analysis, reporting 24 unrelated cases involving eight chromosomes. In conclusion, we observed three different scenarios: true translocation, benign variant and complex rearrangement. The detection of translocation partners is essential to evaluate possible euchromatic unbalances and to infer their effect on phenotype. Moreover, we emphasize the importance to perform both, molecular and conventional cytogenetics methods, to better understand the behavior of our genome.

**Keywords:** cytogenomic instability; acrocentric chromosomes; satellited non-acrocentric chromosomes; chromosome analysis; array CGH

## 1. Introduction

Nearly half of the human genome is represented by repetitive elements whose role remains largely unknown. Recent findings have brought these sequences into the spotlight of genome organization and disease development [1,2]. Dumbovic et al. [2] reported that 56% of the human genome was repetitive DNA, but other studies indicated higher percentages up to 69% [3]. Due to their intrinsic nature of repetitive elements, their presence may cause reciprocal or nonreciprocal translocations, segmental duplications, gene amplifications and other kinds of spontaneous chromosomal rearrangements that ultimately leads to genomic instability, a hallmark of many cancers. In addition, they can induce constitutive chromosome abnormalities which underlie congenital human diseases.

Repetitive DNA can be classified into two large families, "tandem repeats" and "interspersed repeats." Each of these two families can be itself divided into subcategories. Interspersed repeats include interspersed repeats containing transposons, transfer RNA (tRNA) genes and gene paralogues, whereas tandem repeats comprise tandem repeats containing ribosomal DNA (rDNA) repeat arrays and satellite DNA [2]. Satellite DNA is found in heterochromatin regions such as telomeres and centromeres, but also in the short arms of five human autosomes, 13, 14, 15, 21 and 22, identified as acrocentrics. Indeed, all 10 acrocentric chromosomes are recognized by the presence of short satellited arms that contain three bands: p11, p12 and p13. Bands p11 and p13 are composed of satellite sequences, including satellite III (sat III) and β-satellite, packaged as heterochromatin. Band p12 contains approximately 400 copies of the 43 kb rDNA repeat, containing the 18S, 5.8S and 28S rDNA subunits [4]. These rDNA clusters coincide with the chromosomal context around which nucleoli form, termed the nucleolar organizer region (NOR), and with the secondary constrictions of metaphase acrocentric chromosomes [5]. Distal and proximal sequences to rDNA repeat arrays are conserved among the acrocentric chromosomes, suggesting them as sites of frequent recombination. Proximal sequences are almost entirely segmentally duplicated, similarly to the regions bordering centromeres. In contrast, the distal sequence is predominantly unique to the acrocentric short arms and is dominated by a very large inverted repeat [6].

Robertsonian translocations (whole arm exchanges between acrocentric chromosomes) are the most common and recurrent structural chromosome rearrangements (1 in 1000 individuals) [7]. Robertsonian translocations are commonly found in two different genomic scenarios: as an evolutionary rearrangement involved in mammalian karyotypic evolution; and as a chromosomal abnormality with clinical/polymorphic meaning [8]. Interestingly, comparative cytogenomics evidenced that evolutionary breakpoint regions are enriched with different types of repetitive sequences such as SINEs, LINEs, LTRs, segmental duplications and rDNA repeat arrays. In particular, several studies have highlighted that the location of rDNA clusters can rapidly change through transposition [9,10]. Therefore, the rDNA clusters show several features in common with breakpoint regions: they are tandemly repeated; they are generally located in pericentromeric and subtelomeric regions; they transpose; they are subjected to high rates of intra- and inter-chromosomal recombination [9]. Regarding the clinical/polymorphic meaning, loss or gain of the short arm of acrocentric chromosomes occurs without apparent phenotypic effect: in fact, the balanced form of Robertsonian translocations have no associated phenotype.

Chromosomal rearrangements involving the short arms of acrocentric chromosomes are a well-known form of chromosomal variation [11,12]. In addition, translocations between the short arm of an acrocentric chromosome and the heterochromatic region of the long arm of the Y chromosome, resulting in satellited Y chromosomes, are commonly described as normal variants since 1995 [13]. Non-acrocentric chromosomes with terminal satellites (ps, qs) have been described very rarely and revised by Sarri et al. [14]. They arise from a translocation between the short arm of an acrocentric chromosome and the terminal region of another one. Balanced carriers of a translocation are generally unaffected. Contrariwise, individuals with unbalanced rearrangements, with loss of material from the non-acrocentric chromosome, are associated with an abnormal phenotype.

We revised eight ps and qs chromosomes, detected in individuals as de novo or transmitted in families, in order to: infer their effect on phenotype; verify the implication of homologous sequences in the rearrangements involving the partner chromosomes; possibly understand the causative mechanism. In addition, we reported two acrocentric chromosomes with a double satellited region one in canonical position and one in qtel (14qs;21qs).

Finally, we identified a mosaic condition of a jumping satellite between chromosomes 13 and 14 confirming the instable activity of all the acrocentric p arms, also in cases of normal phenotype and overall unchanged genomes.

## 2. Case Reports and Cytogenetics—Genomics Description

### *2.1. 4qs: 2 Unrelated Cases*

#### 2.1.1. Case 1

Cytogenetic evaluation of a couple with history of fetal hydrocephaly was requested. The couple decided to interrupt the pregnancy at 23 weeks of gestation and no fetal tissue was available for karyotyping. Chromosome analysis of father's lymphocytes showed satellites on the long arm of one chromosome 4 (4qs) and the presence of one chromosome 21 without cytological satellites, both inherited from his mother. The 4qs was AgNor positive and C bands negative; fluorescence in situ hybridization (FISH) analysis did not provide evidence of centromeric $\alpha$-satellite sequences of chromosomes 13–21 and 14–22 in 4qtel position, whereas a positive signal of β-satellite sequences was shown. Negative DA-DAPI staining allowed to exclude material derived from chromosome 15.

Figures 1A–C demonstrates that the chromosome 4 was complete and the translocation involved the pan-telomeric sequence of 4 and the β-satellite sequence of 21. In conclusion, a reciprocal translocation between 4q and 21p, without any transfer of 4q specific coding sequences on 21p arm, occurred.

#### 2.1.2. Case 2

Prenatal diagnosis was requested for positive bi-test (increased risk of trisomy 13 and 21). An apparent 8p deletion was observed in fetal chorionic villus cultures. Thus, parents' peripheral blood karyotypes were investigated in order to verify if it could be the result of a balanced translocation. A 4qs was evidenced in the maternal karyotype but was not present in the fetal one. FISH analysis with specific subtelomeric probes showed the retention of specific telomeres; β-satellite sequences were also present in 4qtel. A signal, weaker than the ones located on other acrocentric chromosomes, was detected also on one 14p. In two out of 21 examined metaphases, the 4qs was in association with acrocentric p-arms. Moreover, the observation of bidimensional interphase nuclei after FISH with a β-satellite probe, identified that more than 50% of them presented two brilliant signals and a diffuse pulverization of minute signals, and only 20% showed multiple signals of discrete dimensions, demonstrating a normal nucleolar activity (data not shown).

### *2.2. 5qs: 1 Familiar Case*

The karyotype of the couple was requested for medically assisted procreation for infertility. The male karyotype was normal whereas the woman's karyotype showed one chromosome 5 with satellites at q arm (Figure 1D). Her father resulted to be carrier of the same 5qs.

FISH analysis with specific subtelomeric probes showed correct signals on both chromosomes 5 (Figure 1E). The exchange occurred between the telomeric repeats of chromosome 5 and the DNA satellite repeats of one not identified acrocentric chromosome. The segmental duplications of telomeres 5q showed paralogue sequences in 21p11.1 and 21q11.2 regions (Figure 8), indicating that they may have mediated a pairing as intermediary of exchange. In 7% of the examined metaphases association with acrocentric p-arms was observed.

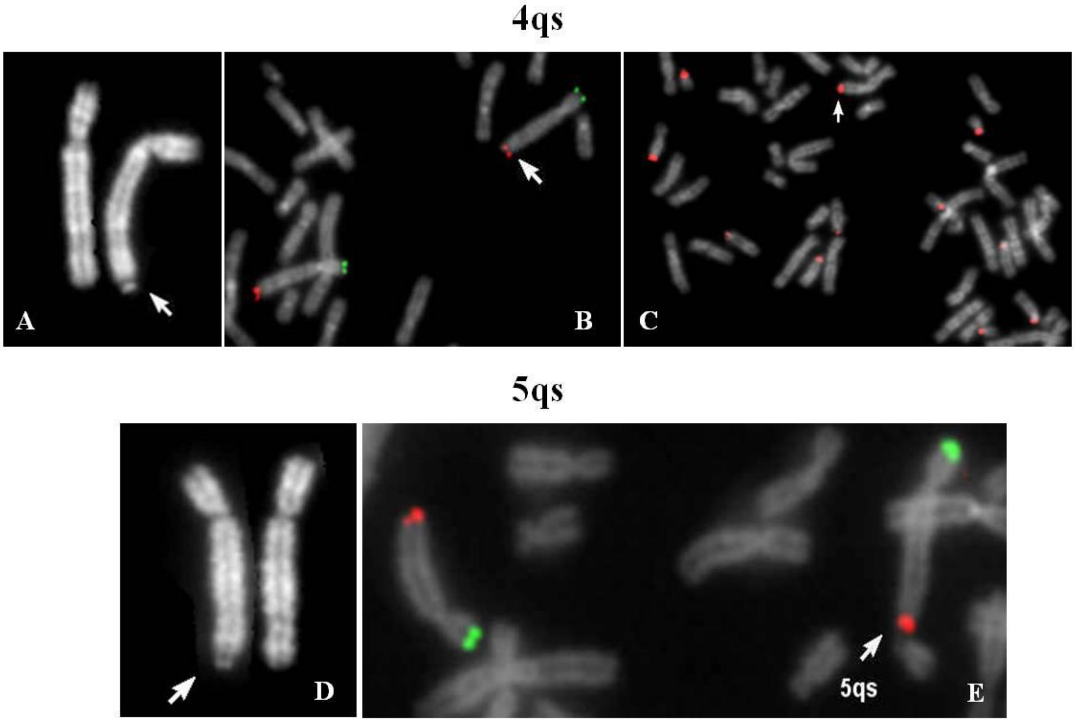


**Figure 1.** 4qs case 1: (**A**) Quinacrine banding (QFQ) (4qs is arrowed); (**B**) specific subtelomeric probes of chromosome 4 (green p-arm, red q-arm); (**C**) β-satellite probe (arrow indicates the 4qs chromosome). 5qs: (**D**) QFQ banding (5qs is arrowed); (**E**) specific subtelomeric probes of chromosome 5 (green p-arm, red q-arm) (arrow indicates the 5qs chromosome associated with chr 21). All images were captured at 100× magnification (see materials and methods for details).

### *2.3. 7qs: 1 Case*

A 4 year-old female proband was analyzed because of psychomotor retardation and facial dysmorphisms. Conventional cytogenetic analysis showed that one chromosome 7, longer than normal, had q arm with satellites (Figure 2A) and that the maternally derived chromosome 22 presented a new polymorphism (Figure 2B). The parental karyotypes were normal.

FISH with β-satellite probe showed signals on one 7qter and all acrocentric chromosomes except for one chromosome 22 (Figure 2D). FISH with specific subtelomeric probes evidenced the lack of signal on one 7q (Figure 2C), whereas the probe for all common human telomeres showed signal at the satellite of 7qs (not shown). The absence of signals for the common $\alpha$-satellite probe and the specific 13/21 and 14/22 centromeric probes was also observed (not shown). The whole chromosome 7 painting showed no hybridization for the chromosomal satellite of 7qs (Figure 2E). Array CGH (Figure 2F) allowed us to define the entity of the abnormality on the 7q region: arr[GRCh37] 7q35q36.3(144500074_158924090)x3,7q36.3(159043902_159118707)x1. De facto this anomaly results in an inverted-duplication (invdup?) as described for 8p [15–17]. The deleted trait is reported in database DGV (Database of Genomic Variants, http://dgv.tcag.ca/dgv/app/home). Moreover, no homology with other chromosomes was detected (Figure 8).

The female proband was born at 39 weeks of gestation, with caesarean section for breech presentation after an uneventful pregnancy. APGAR scores were 8 and 10 after 1 and 5 min, respectively. Low neonatal birth weight (1920 gr), and transient hypocalcemia were reported. Cyanosis and echocardiographic evidence of interatrial communication, such as ostium secundum atrial septal defect, appeared on the fourth day.

She started sitting at 7–8 months and walking at 18 months. No problems of sleep or feeding, but several episodes of respiratory infections in the first year of life (bronchiolitis and spastic bronchitis) were reported. At 2 years old, she pronounced different phonemes (not words), showed

poor concentration in the game (15′ maximum), discrete manual skills, good socialization with other children, differentiated reactions with adults and good understanding.

The morphologic evaluation identified synophrys (familial), modest turricephaly, retracted auricles, stuck helix, hypoplastic lobule, single palmar groove in the right hand and clinodactyly of the fifth finger of the left hand.

Neuropsychiatric evaluation at 3 years old, showed that she had a good understanding of simple deliveries but did not easily come into contact with the examiner. The verbal language was characterized by the presence of dyslalia. Ligamentous laxity and modest global motor embarrassment were also present.

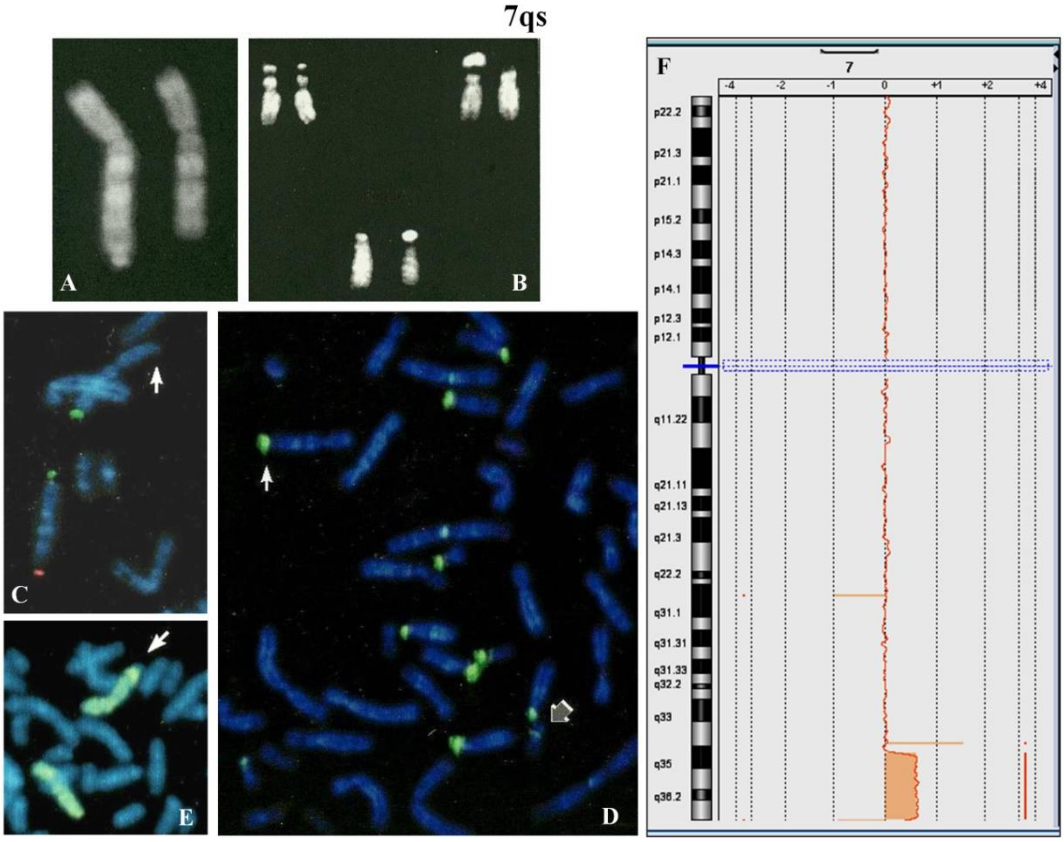


**Figure 2.** 7qs: (**A**) QFQ banding of chromosomes 7 (7qs on the left); (**B**) chromosomes 22 of father (left), mother (right) and proband (below); (**C**) partial metaphase after FISH with specific subtelomeric probes (green p-arm and red q-arm). Red signal is absent on one chromosome 7 (arrow). (**D**) FISH with β-satellite probe: the white arrow indicates the positive q-arm of 7qs; the gray arrow a chromosome 22 with weak signal. (**E**) partial metaphase after FISH with whole chromosome 7 painting, the arrow indicates the rearranged 7 showing the terminal q region without hybridization. All images were captured at 100× magnification (see materials and methods for details). (**F**) array CGH showing the larger duplicated and the small terminal deleted regions. The other 2 proximal variants are described as benign copy number variation (CNV) (Database of Genomic Variants, http://dgv.tcag.ca/dgv/app/home).

## 2.4. *9qs: 1 Familiar Case*

The chromosome 9qs was identified in a fetus after amniocentesis requested for advanced maternal age (35 years, 2 pregnancies, 1 delivery).

The investigation extended to other family members, evidenced the same chromosome 9qs in the father (Figure 3A) and in the previous child, who undergone surgery for a modest atrio-ventricular defect at 1 year old. The father had another child with normal karyotype with a different partner. The paternal uncle was a carrier of the same 9qs and his wife had multiple miscarriages.

FISH analysis showed signals with 9q specific probe on both chromosomes 9 in normal location (Figure 3B).

All chromosomes of groups D (13, 14, 15) and G (21, 22, Y) showed cytological stalks and satellites, but in all the metaphases evaluated in both brothers no association of 9qs with acrocentric p-arms was observed, even in cultures set up years later. A very high homology segmental duplication sequence was present on 9qter and 22q11.21 (Figure 8).

### *2.5. 12ps: 1 Case*

A 31 year-old woman with three problematic pregnancies was identified. The first pregnancy resulted in the birth of a female who died after 2 h for a severe diaphragmatic hernia. The second one evolved into a spontaneous abortion at the 15th week of gestation and the third had a prenatal diagnosis of trisomy 21 followed by induced abortion. High resolution G banding analysis identified a reciprocal translocation between the p arms of chromosomes 12 and 14: t(12;14)(p12–13;p11) (data not shown). A consensus for a deeper study was not acquired.

### *2.6. 17ps and 17qs: 2 Unrelated Cases*

#### 2.6.1. Case 1

A woman requested prenatal diagnosis because of advanced age (36 years old) and fetal evidence of cystic hygroma with increase nuchal translucency (value 7.6) observed at $13^{th}$ week of gestation. After chorionic villus sampling, the analysis of cytotrophoblasts (direct analysis) showed a normal male karyotype.

At the same time Array CGH analysis on placental biopsy was performed and a segmental trisomy of 17p chromosome was evidenced: arr[GRCh37] 17p13.3p12(1693_13611322)x3 (Figure 3C). FISH study on the cytotrophoblasts revealed that the trisomic 17p region was translocated on one chromosome 15p (Figure 3D). In the maternal karyotype a balanced translocation between 17p and 15p was identified (Figure 3E) and confirmed by FISH (Figure 3F). FISH with β-satellite probe showed a positive signal on the p-arm of the derivative 17 and the absence of signal on the derivative chromosome 15 (Figure 3G). In 9% of the examined metaphases, association with acrocentric p-arms was observed.

At 15th week of gestation, the pregnancy was interrupted and persistence of cystic hygroma was observed.

The woman had two previously pregnancies: the first, a twin pregnancy, was spontaneously aborted in the first trimester (no cytogenetically investigated); the second one had ended in a phenotypically normal female. In both cases karyotype analysis was not performed.

#### 2.6.2. Case 2

The karyotype of the couple was requested in the context of medically assisted procreation. The husband had a normal karyotype, whereas the wife (35 years old) showed a chromosome 17 with a satellite at q25.3. Eleven percent of metaphases showed association of the 17qs with acrocentrics (data not shown). Further analyses were not performed.

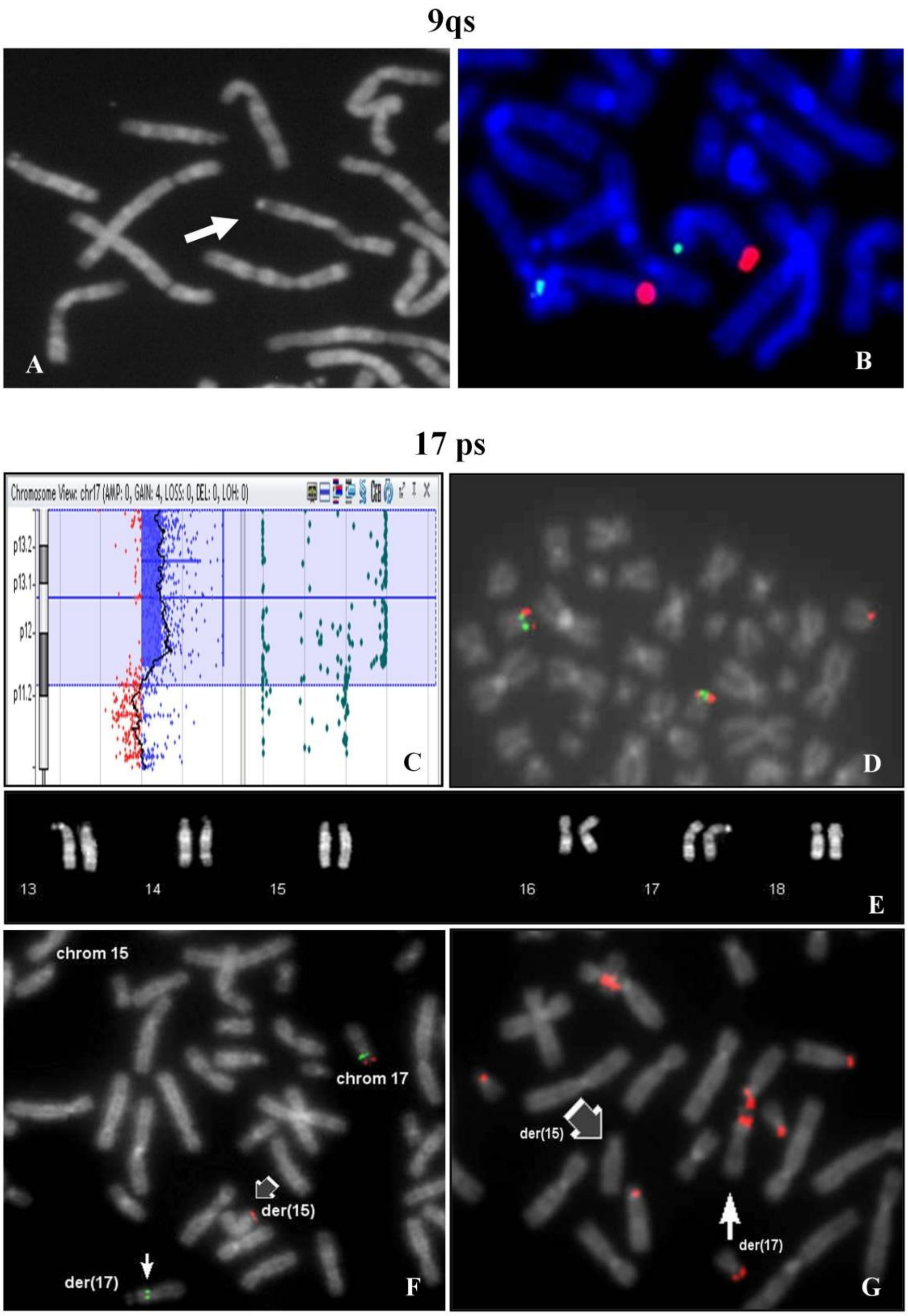


**Figure 3.** 9qs: (**A**) partial metaphase showing the 9qs of the father (arrow); (**B**) specific subtelomeric probes of chromosome 9 (green p-arm, red q-arm). 17ps: (**C**) array CGH showing the segmental trisomy of 17p; (**D**) FISH on partial metaphase of the fetus (direct analysis) with LIS1 specific probe (17p13.3, red signals) and FLI specific probe (17p11.2, green signals). The trisomic 17p region was translocated on chromosome 15p (red signal). (**E**) QFQ banding of 13, 14, 15, 16, 17 and 18 maternal chromosomes; (**F**) FISH on partial metaphase of the mother with LIS1 probe (17p13.3, red signals) and FLI probe (17p11.2, green signals). A balanced translocation between 17p and 15p was identified (single green and red signals). (**G**) FISH on partial metaphase of the mother with β-satellite probe: the white arrow indicates the positive p-arm of derivative 17 (in association with one chromosome **D**) and the gray arrow the negative derivative chromosome 15. All images were captured at 100× magnification (see materials and methods for details).

### *2.7. 20ps and 20qs: 4 Unrelated Cases*

#### 2.7.1. 20ps Case 1

Amniocentesis was requested at 17th week of gestation because of advanced maternal age (39 years). All the analyzed metaphases showed a chromosome 20 with a chromosomal satellite on p arm. All the other chromosomes showed a normal morphology. The paternal karyotype was normal, while the maternal one presented the satellite on chromosome 20. FISH analysis with β-satellite probe on maternal lymphocytes evidenced no signals on 20ps, but positive signals on all the acrocentrics, except one (group D). FISH analysis using the specific subtelomeric probes of chromosome 20 revealed the presence of the signals in the correct positions (data not shown).

The conclusion was that a p acrocentric arm was translocated on a chromosome 20 without reciprocal exchange (half translocation?). The rearrangement involved only repeat sequences and the prognosis for the pregnancy was good. The baby was delivered at term and healthy.

#### 2.7.2. 20ps Case 2

Karyotype analysis for male infertility in assisted procreation program evidenced an association of 20ps with all acrocentric chromosomes in 20% of the analyzed cells (data not shown).

#### 2.7.3. 20ps Case 3

Karyotype analysis in a couple that required prenatal diagnosis evidenced a chromosome 20 with satellite in telomeric p arm in the male. Association of 20ps with all acrocentric chromosomes was observed in 18% of the investigated cells (data not shown).

#### 2.7.4. 20qs

A pregnant woman, after four pregnancies, but only two deliveries, requested prenatal diagnosis for maternal advanced age (39 years). Amniocentesis was performed at $16^{th}$ week of gestation. Fetal karyotype showed a rearranged chromosome 20 with brilliant satellites on q arm (Figure 4A,C), negative after DA-DAPI staining. The parental karyotypes were normal. In the paternal one, a polymorphic brilliant DA-DAPI negative satellite was observed on a chromosome 22 (Figure 4B,D).

After the QFQ polymorphism study, it was evident that the fetus did not inherit the polymorphic paternal chromosome 22. It was supposed that in the fetus the paternal 22 variant was involved in a translocation with a chromosome 20. FISH analysis with the β-satellite probe showed that the 20qs was negative (Figure 4F) and the WCP20 probe did not hybridize on the satellite of the der(20) (Figure 4H). All human telomeres and chromosome 20 specific subtelomeric probes (Figure 4E,G) confirmed the hypothesis of a balanced translocation between chromosome 20 and chromosome 22. A normal female was delivered at term.

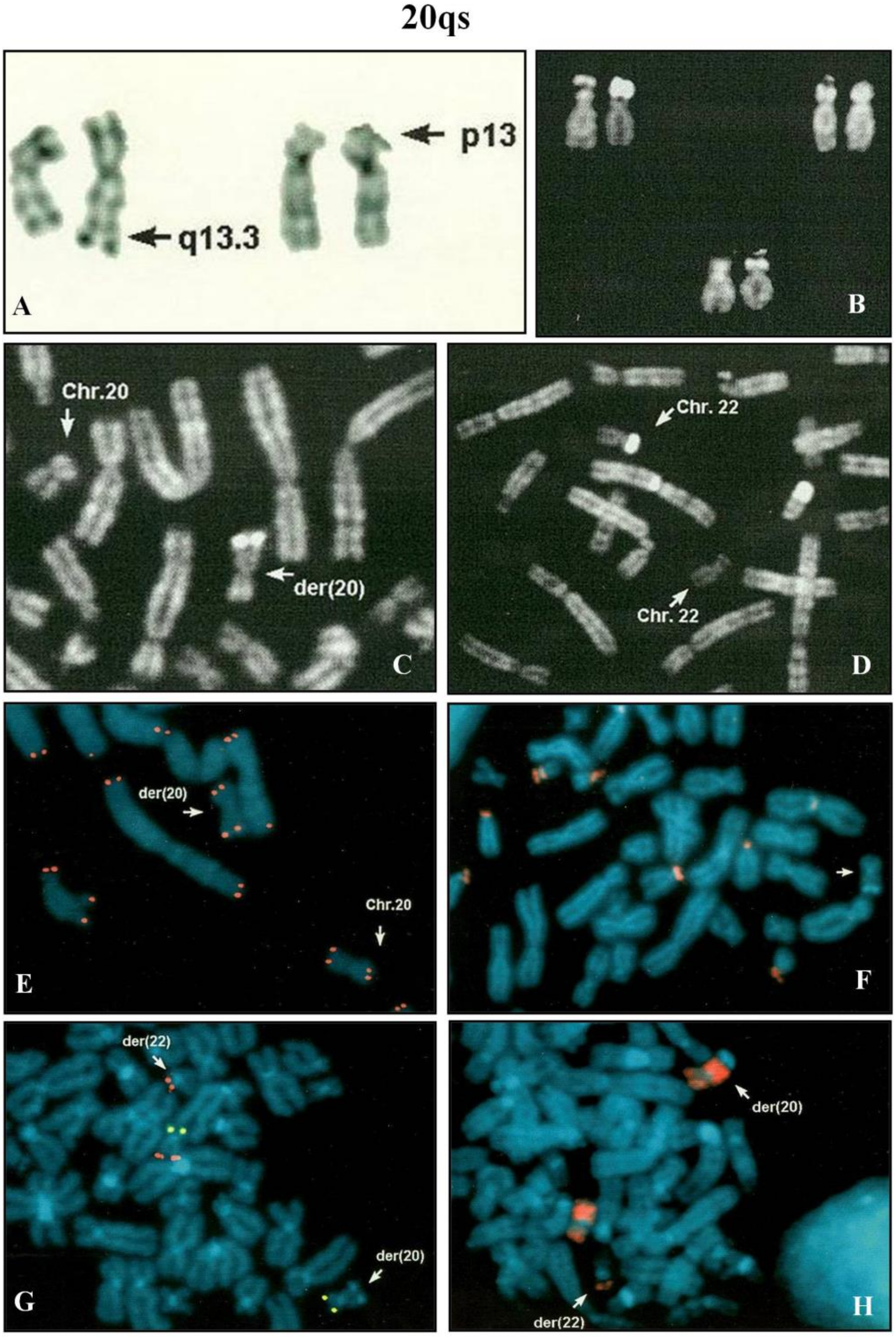


**Figure 4.** 20qs: (**A**) reciprocal translocation involving a chromosomes 20 and a chromosome 22 (GTG banding); (**B**) chromosomes 22 of whole family: father (left), mother (right) and fetus (below) showing the brilliant 22 satellites in the father karyotype, absent in fetus because translocated on 20q; (**C**) partial fetal QFQ banded metaphase with both chromosomes 20 arrowed; (**D**) partial paternal QFQ banded metaphase with both 22 arrowed; (**E**) FISH on fetal metaphase with pan-telomeric probe marked all chromosomes, included the der(20); (**F**) FISH with β-satellite probe evidenced both chromosome 22 with hybridization signals, while the der(20) was negative (arrow); (**G**) partial fetal metaphase after FISH with specific subtelomeric probes of chromosome 20 (green p-arm and red q-arm). Subtelomeric 20q red signal was on derivative chromosome 22p arm; (**H**) partial fetal metaphase after FISH with whole chromosome 20 painting: wcp20 painted all der(20), but not the satellited region, small signals of hybridization were also evident on 22 p arm. All images were captured at 100× magnification (see materials and methods for details).

### *2.8. 14qs: 1 Case*

A chromosome 14qs was identified in the karyotype of a 44 year-old woman, investigated after repeated spontaneous abortions (Figure 5A). All acrocentric chromosomes showed the p arms stalk and satellited (Figure 5B) with the exception of the 14qs, which was never observed in association with the other acrocentrics.

### *2.9. 21qs: 1 Case*

Genetic tests were requested in the context of medically assisted procreation for infertility. The female's karyotype showed a 21qs (Figure 5C). The presence of specific subtelomeric sequences preserved the integrity of chromosome 21 (Figure 5D). It was, however, not possible to determine the origin of the supernumerary satellites. Both satellites of 21qs (p arm and q arm) were associated with all acrocentric chromosomes (29% of metaphases). All acrocentric chromosomes presented stalks and satellites in the correct position.

### *2.10. Satellite Jumping: 13ps vs 14ps*

A 33 year-old female, tested for repeated spontaneous abortions, presented a normal karyotype with a mosaic situation regarding a big and brilliant chromosomal satellite, jumping between chromosomes 13 (10 metaphases) and 14 (6 metaphases) (Figure 5E,F). The chromosome polymorphisms were concordant between the two cell lines and the contamination with other contemporaneous karyotype analyses was excluded.

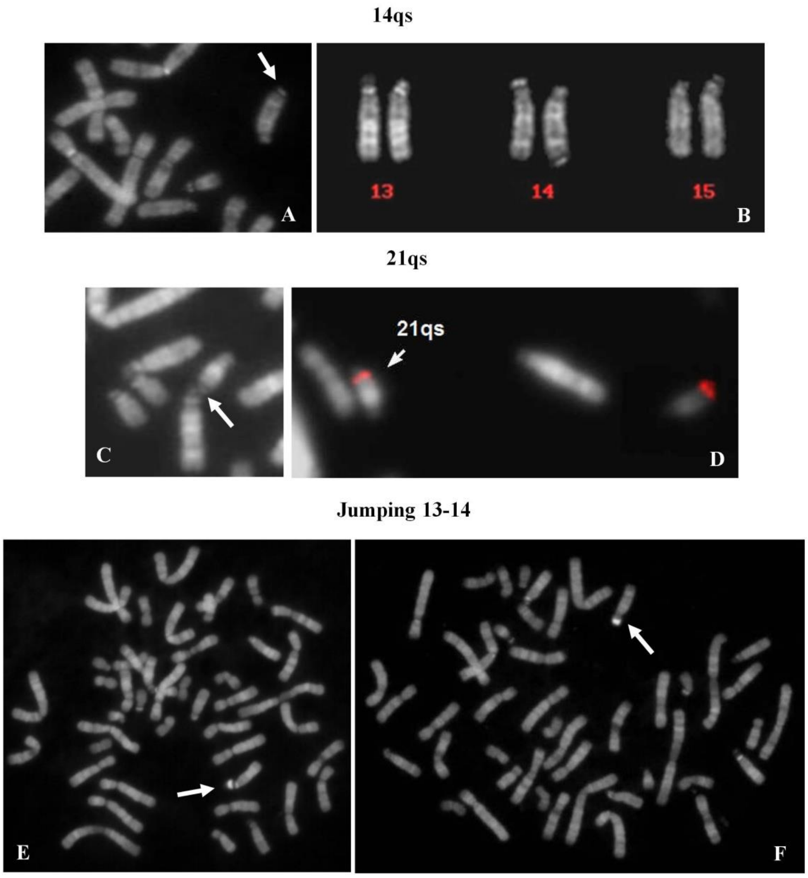


**Figure 5.** 14qs: (**A**) partial metaphase showing the 14qs (arrow); (**B**) chromosomes 13, 14 and 15: one chromosome 14 showed the cytological satellite on q arm. 21qs: (**C**) partial metaphase showing the 21qs (arrow) in association with chr 14; (**D**) specific subtelomeric probes of 21q hybridized both chromosomes 21. Satellite jumping: metaphases showing the satellite jumping between (**E**) chromosome 13 and (**F**) chromosome 14. All images were captured at 100× magnification (see materials and methods for details).

*2.11. Yqs: 12 Unrelated Case (2 Ynfqs) (1 invqs)*

Group 1—Five cases were identified in the context of medically assisted procreation for infertility. All Yqs were β-satellite positive after FISH (Figure 6A,B). One of these showed a QFQ banding non-fluorescent Y chromosome, but a brilliant band after netropsine–DAPI staining was evident (Figure 6C,D) [18]. Group 2—Two males, one of which carrier of an inverted chromosome Y with satellites (Figure 6E,F), were tested because of repeated spontaneous abortions. Case 3-A Y chromosome without brilliant heterochromatin was observed in an adult male, who was investigated for a previous fetus showing a not better referred chromosomal abnormality (data not shown). Case 4—One case was detected in fetal cells. The father karyotype was investigated (data not shown). Group 5—Three cases were detected in males studied for different causes: hypostaturalism/hypoevolutism, mental retardation without a defined diagnosis and an unspecified chromosome anomaly in a consanguineous (data not shown).

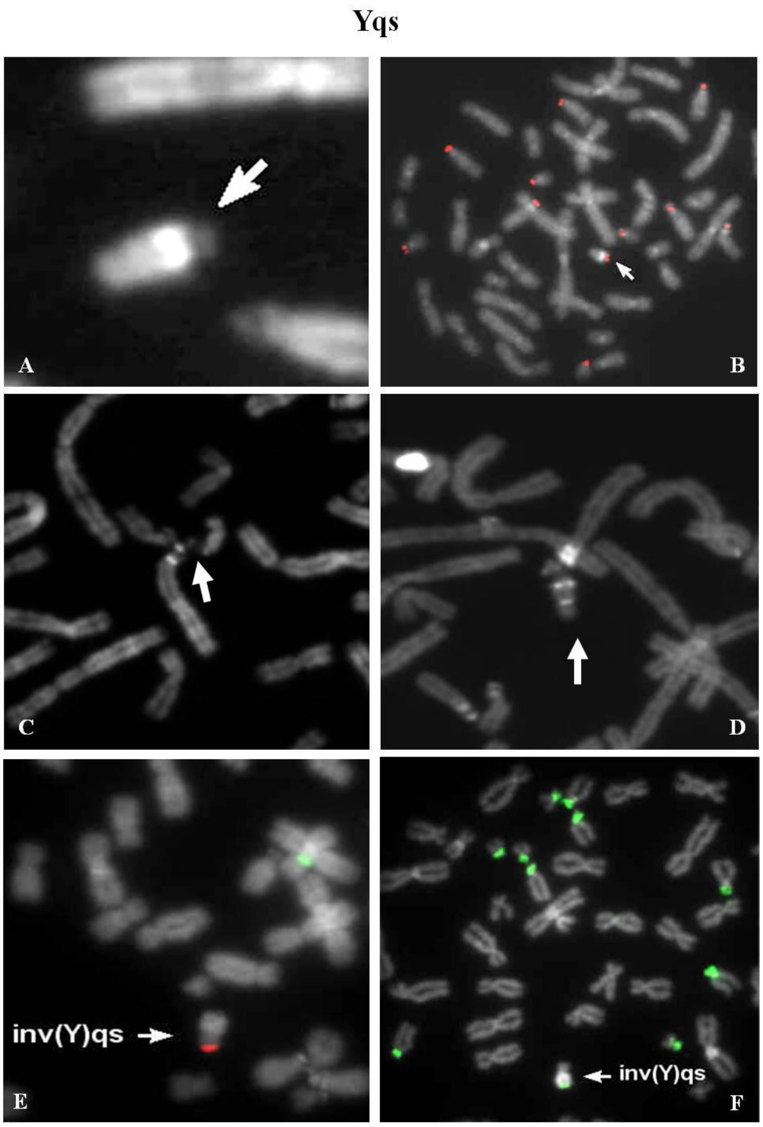


**Figure 6.** Yqs: (**A**) partial metaphase showing the satellited Yq (arrowed); (**B**) β-satellite probe signal on Yqs; (**C**) QFQ-banded Yq satellited chromosome with loss of heterochromatic region (Yqh⁻); (**D**) netropsine–DAPI staining showed the presence of a small heterochromatic region on chromosome Yqh⁻ satellited. Both metaphases (**C**,**D**) showed Yqs–acrocentrics association; (**E**) FISH on partial metaphase with SRY/CEPX probe (red and green signals): the chromosome inv(Y)(p11.2q11.23)qs showed the SRY signal in the canonical position; (**F**) FISH on partial metaphase with β-satellite probe evidenced hybridization signal at the end of the heterochromatic region of inv(Y)qs. All images were captured at 100× magnification (see materials and methods for details).

### *2.12. Causative Mechanisms of ps–qs*

Different mechanisms are involved in ps–qs formation in our cases. In most cases the telomeric region of a chromosome may were involved in a non-allelic homologous recombination (NAHR) event with the short arm of an acrocentric chromosome (Figure 7A). NAHR is mediated by the presence of homologous repetitive sequences, such as β-satellite sequences, segmental duplications or LINE and SINE elements [19].

After this rearrangement, rDNA cluster and β-satellite sequences may were translocated on another chromosome (Figure 7B). This mechanism could define the 4qs (case 2.1.2), 5qs, 17qs, 20ps (case 2.7.2), 20ps (case 2.7.3) and 21qs cases. The presence of rDNA may explain the association of ps–qs chromosomes with other acrocentric chromosomes evidenced in some metaphases. For this reason, association is characteristic only for short arms of acrocentric chromosomes. rDNA clusters coincide with the NORs and this association can be observed in normal chromosome preparations (Figure S1).

In other cases, rDNA cluster remains on the acrocentric chromosome and only β-satellite sequences translocate (Figure 7C). This mechanism could describe the 4qs (case 2.1.1), 9qs and 14qs and it may were mediated by β-satellite sequences or segmental duplications (Figure 8).

In three cases (12ps, 17ps and 20qs) the ps–qs chromosomes were the result of a true translocation event. A 7qs-case was a complex rearrangement with a terminal deletion and a duplication of the subtelomeric region (invdup?, Figure 7D). In addition, a satellite at the common telomere of the rearranged q region was present, probably due to a mechanism that stabilized the invdup del(7q) through telomere capture of 22p [20]. Finally, Yq did not show homologous repetitive sequences that may explain the Yqs formation. Moreover, the entire Y chromosome was reported to associate with nucleoli in a high-resolution analysis [21]. This could have led to interaction with acrocentric chromosomes.

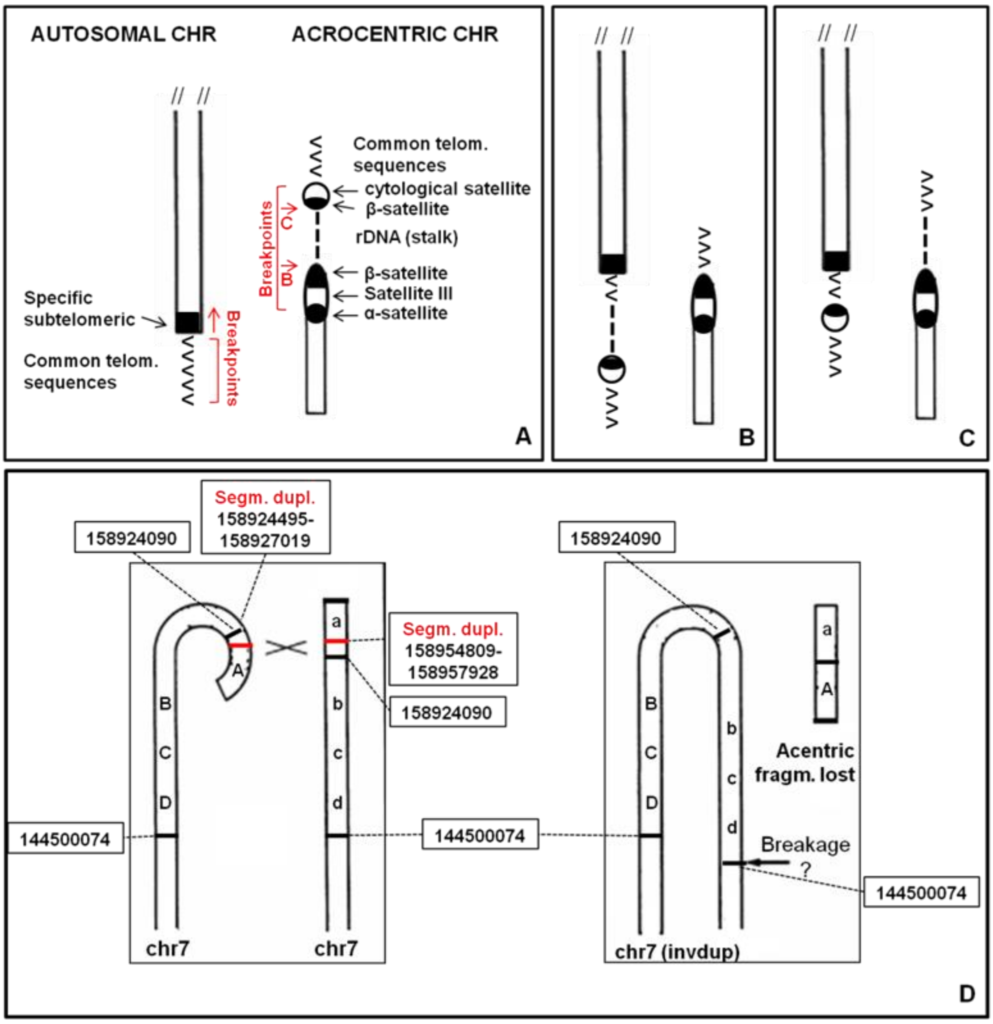


**Figure 7.** Causative mechanisms of ps–qs formation. (**A**) homologous repetitive sequences in telomeric regions and acrocentric chromosomes; (**B**) first type of non-allelic homologous recombination (NAHR) (cases 4qs, 5qs, 17qs, 20ps, 20ps and 21ps); (**C**) second type of NAHR (cases 4qs, 9qs and 14qs); (**D**) 7qs complex rearrangement (invdup?) mediated by segmental duplications. Black lines: breakpoints, red lines: segmental duplications.

## 3. Discussion

In the cytogenomic era classical cytogenetics provides important data. The use of both molecular techniques and morphologic analyses led to deeply understand our genome "behavior", providing good diagnoses. We have already reported that the instability of the short arm of acrocentric chromosomes is well known [22]. Non-acrocentric chromosomes with satellites have also been described [13]. In fact, we have previously reported cases of cytological satellites in non-classical positions (1qs in a family, a case of 14qs) [23,24], presenting homologous sequences between p arm of acrocentrics and recipient chromosome/region.

The data presented here take into account a larger number of cases, highlighting two important aspects: (i) acrocentric p arm breakpoints are variable and located along the whole p length; they identify a very unstable region, never involving the centromeres; and (ii) some ps or qs autosomal chromosomes display association with acrocentric chromosomes. Therefore, they must have received rDNA, whereas the others received only the cytological satellite (Figure 7A–C).

Ribosomal DNA participates in the dynamic process of nucleoli formation with telomeres and β-satellite sequences [6,25,26]. The β-sequences are mapped not only on the p arm of acrocentric chromosomes, but also scattered in several autosomes, as displayed in Figure 8 [27–29].

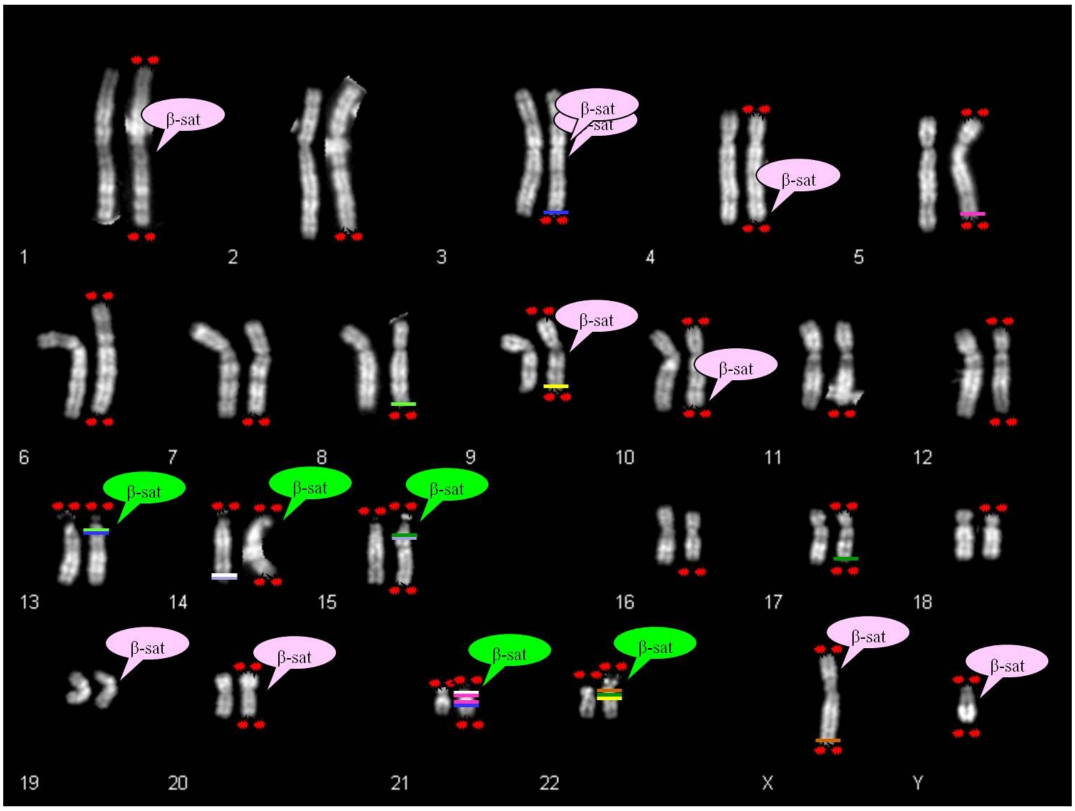


**Figure 8.** β-Satellite clusters DNA mapping [27–29] in a human normal male karyotype. Red spot: satellites on the short and long arms of chromosomes; for non-acrocentric chromosomes satellites are collected by literature or our cases. Green cloud: canonical beta-satellite sequences on acrocentric chromosomes; pink cloud: interspersed beta-satellite sequences on non-acrocentric chromosomes. Colored lines indicate homologous segmental duplications between telomeric regions of non-acrocentric chromosomes (except for 14qs case) and p-arms of acrocentric chromosomes; homologous tracts are shown with a line of the same color and precisely: 3q29 homologous with 13q12.11 and 21q11.2 (blue line); 5q35.3 homologous with 21p11.1 and 21q11.2 (fuchsia line); 8q24.3 homologous with 13q12.11 (fluorescent green line); 9q34.3 homologous with 22q11.21 (4 consecutive traits in the same cytogenetic band)(yellow line); 14q32.33 homologous with 15q11.1 and 15q11.2 (lilac line); 14q32.33 homologous with 21p11.2 (white line); 17q24.3 homologous with 15q11.2 and 22q11.21 (dark green line); Xq28 homologous with 22q11.1 (light brown line).

There is a partial correspondence between the distribution of β-sequences and satellited autosomes. In fact, it was observed that the chromosomes 2, 5, 6, 7, 8, 11, 12, 16, 17, 18 do not show β-sequences despite ps–qs chromosomes were reported. Moreover, β-sequences are clearly proximal to centromeres in chromosomes 1, 3, 9 and 20, and cases of satellited chromosomes have also been described. To date, there is only one chromosome (chr 19) without the description of chromosomal satellite, despite the presence of β-sequences.

The search in UCSC Genome Browser for segmental duplications in autosomal telomeric regions led to the identification of some homologies in pericentromeric regions of acrocentric chromosomes. These autosomes do not show β-satellite sequences in their DNA content. For example, the chromosome 5 shows segmental duplications homologous to two regions of chromosome 21 (21p11.1; 21q11.2). Likewise, chromosome 17 shows telomeric segmental duplications homologous to chromosomes 15 and 22. Chromosome 9 displays pericentromeric β-sequences, but also homology between q telomere and 22q11.21 region (Figure 8).

However few things still remained unexplained. For instance, our 7qs case and the 10qs described by Sarri et al. [14] do not have β-sequences, neither segmental duplications homologous to acrocentric chromosomal sequences. Both cases are complex rearrangements with a deletion of the telomeric region and a duplication of the subtelomeric one (invdup?). In addition they have satellite

at the common telomere of the rearranged q region, probably due to a mechanism that stabilized the invdup.

In terms of phenotype, three different scenarios were identified ((confirmed also in published cases [14]): i) true translocation (12ps, 17ps, 20qs); ii) normal "benign" variant (4qs, 5qs, 9qs, Yqs); iii) complex rearrangement (7qs).

In true translocation the autosomal satellited recipient shows an interstitial breakpoint (above the telomeric region). Thus, the loss of specific telomeric sequences is observed. The acrocentric donor chromosome holds an euchromatic region derived from the autosomal partner. The phenotype can be either normal or abnormal, based on the balanced or unbalanced status of the translocation.

In case of "benign" variant, the rearrangement involves the common telomere sequences of the recipient chromosome. Conversely, the specific telomeric sequence remains at the natural location. The rearrangement involves only the repeated sequences also in case of acrocentric donor chromosome (Figure 7A–C). Subjects carrying this type of translocation show a normal phenotype. One or both derivatives can be present in a karyotype. They can be independently inherited by subsequent generations, without any apparent selective pressure.

We have recently identified two families with normal phenotypes, in which FISH analysis, with the specific subtelomeric probes of chromosomes 4q and 7q, evidenced a third signal at 13p11.2, indicating the presence of a trisomic segment [22]. Being known the regulatory role of heterochromatin in the transcription silencing [30,31], the localization of a segmental trisomy on the short arm of an acrocentric chromosome could explain the lack of a phenotypic effect in almost two generations of both families. It is noticeable that these variants are not identifiable by molecular karyotype.

In case of complex rearrangement, the loss of the specific telomeric region of the non-acrocentric satellited autosome is observed. The recipient appears to be involved in more than one breakage. The phenotype is abnormal.

At our knowledge, all cases of ps–qs showing pathologic phenotypes, described in literature and in this study, originated from unbalanced derivatives of balanced translocations, both de novo and familial, with the exception of the case reported from Sarri et al. [14] and our case 7qs, that are complex rearrangements (Table 1).

**Table 1.** Summary of cases from literature [14] and described in this work.

| Chromosome Recipient | Chromosome Donor | *n of Cases* (“) | Inheritance | | Phenotype Abnormal/ Normal | n of Cases Balanced/ Unbalanced |
|---|---|---|---|---|---|---|
| | | | De Novo | Fam | | |
| 1 | 13, 15, 21, D, un | 9 | 3 | 5 | 4/3 | 1/5 |
| 2 | un | 11 | 2 | 8 | 1/9 | 1/3 |
| 3 | 15 | 1 | | 1 | 0/1 | 1/0 |
| 4 | 14, 15, 21, 14 or 22, un, 21, un | 16 + 2 | 2 | 11 + 1 | 5/11 + 2 | 4 + 1/11 + 1 |
| 5 | 13, un | 1 + 1 | | 1 + 1 | 1/1 | 0/1 + 1 |
| 6 | 21, un | 2 | | 2 | 1/1 * | 1/1 |
| 7 | 13, un, 22 | 3 + 1 | 1 + 1 | 2 | 1+1/1 | 2/1 |
| 8 | un | 1 | | 1 | /1 | /1 |
| 9 | 13, 22, un | 3 + 1 | | 3 + 1 | 1/2 + 1 | 3/1 |
| 10 | 13, un | 3 | | 3 | 2/1 ° | 1/1 ° |
| 11 | 22, un | 2 | | 2 | | |
| 12 | 21, un, 14 | 7 + 1 | | 7 | 1 **/8 + 1 | 2 + 1/ |
| 13 | | | | | | |
| 14 | un, un | 1 + 1 | | | /1 | |
| 15 | un | 1 | 1 | | 1/ | |
| 16 | 13 | 1 | | 1 | 1/ | /1 |
| 17 | 15, un, 15, un | 2 + 2 | 1 | 1 + 1 | 1/1 | /2 |
| 18 | 13 | 1 | | 1 | /1 | 1/ |
| 19 | | | | | | |
| 20 | D, 22, un, un | 4 | 1 | 1 | /4 | 2/2 |
| 21 | un, un | 1 + 1 | 1 | | 1/1 | /1 |
| 22 | | | | | | |
| X | 21, un | 3 | 2 | 1 | 3 | 1/2 |
| Y | 14, 15, un, un | 24 + 12 | 4 | 18 + 1 | 1 + 2/23 + 10 ° | 4/7 + 12 |
| total excluded Y | | 83 | 15 | 55 | 25/51 | 22/35 |

In “Inheritance” column the remaining cases to reach the total number are unknown. un: unknown; D: chromosome group; M: male; F: female; age: at diagnosis; *: infertility; **: short stature; °: normal phenotype, but unbalanced karyotype; “: one family with 2 brothers and 2 children. Cases presented are in red.

The donor chromosome of the satellites has been identified only in 39% of cases reported in the literature [14] and in those reported here. Among the five acrocentric chromosomes is prominent the role of chromosomes 15 (35% of all the described cases), 13 (23%) and 21 (21%). Regarding the Yqs chromosome, it accounts for 30.2% of cases (Table 1).

In our experience, the clinical indications for the diagnosis were suggestive, because the ps or qs chromosomes were detected in infancy/pediatric age in very few cases and in fetal life in one case, whereas the mean age of diagnosis in adult life was very advanced: from 33 to 47 years (Table 2).

**Table 2.** Summary of cases.

| Chromosome | *n* of Probands | Age of Index Case | Sex | Recipient Arm | Origin of Satellite |
|---|---|---|---|---|---|
| 4 | 2 | 33 | XY, XX | q | 21, 14 |
| 5 | 1 pat | 38 | XX | q | nd |
| 7 | 1 | 4 | XX | q | 22 |
| 9 | 1 * | fetal life | XY | q | nd |
| 12 | 1 | 31 | XX | p | 14 |
| 14 | 1 | 44 | XX | q | nd |
| 17 | 2 | 36; 35 | XX | p, q | 15, nd |
| 20 | 3 + 1 mat | p: 35; 47; 39<br>q:39 | XX, XY | 3 on p,<br>1 on q | D, 22, nd<br>nd |
| 21 | 1 | 32 | XX | q | nd |
| Y | 12 | 15–47 | 12 XY | q | nd |

nd: not determined because impossible to identify; p: p chromosomal arm; q: q chromosomal arm, pat: inherited from the father, mat: inherited from the mother, *: a family composed by 2 brothers and 2 children.

Considering that reproductive problems are the most important and frequent cause of karyotyping request in adult life, the following comes to mind: can these aberrant chromosomes influence somehow the meiotic segregation resulting in an embryo with abnormal karyotype and abortion (couples with repetitive pregnancy interruptions)? Or can they result in a block of meiosis leading to infertility)? To date, we can only speculate as we don't know have data about the real frequency of ps and qs chromosomes in a normal population.

Independently from clinical indications, the frequency of non-acrocentric satellited chromosomes appears to range from 0.02% to 0.05%, considering all the participating laboratories. Sarri et al. [14] has referred a percentage of 0.02% in his department, so the frequency corresponds to our lower limit.

Other studies regarding these cases are needed to better understand the mechanisms producing these rearrangements. In this way, it will be possible to accurately predict what would happen to carriers.

## 4. Materials and Methods

### *4.1. Chromosome Analysis*

The cases were collected from five Medical Genetics Labs. All of these applied the standard methods to conduct the chromosome analysis, following the Italian Society of Human Genetics (SIGU) guidelines [32] and subsequent modifications online, https://www.sigu.net/show/documenti/5/1/linee% 20guida% 20e% 20raccomandazioni?page=1 25 Feb 2014] [33].

In summary, the PHA stimulated lymphocyte cultures were collected after 72 h and the slides were stained with QFQ banding. If necessary, all other types of staining were used (GTG, CBG, AgNOR, DA-DAPI or netropsin-DAPI). Amniocytes were cultured using standard techniques and chromosomal preparations performed both in suspension and in situ.

The karyotype was expressed following the guidelines of the International System for Cytogenomic Nomenclature 2016 (ISCN 2016) [34] using software dedicated to karyotyping: CW4000 Karyo (Leica), Ikaros (MetaSystems), Chromowin (Tesi Imaging). All images were captured at 100X magnification. Some of them were then enlarged photographically.

### *4.2. FISH Analysis*

Fluorescence in situ hybridization analyses were performed according the manufacturer's protocol. In particular, TotelVysion Multi-color FISH Probe kit (Vysis, Abbott Park, Illinois, USA) was used. This include various combinations of TelVysion, CEP and LSI probes specific to single

human chromosome arms and contain loci estimated to be within 300 kb of the end of the respective chromosomes. Probes are labeled in different colors with three spectrally independent fluorophores (SpectrumGreen, SpectrumOrange e Spectrum Aqua).

Moreover, Chromoprobe Multiprobe®-T (Cytocell, Oxford Gene Technology, Cambridge, UK) was used. This device includes subtelomere specific probes for both the p-arm and the q-arm of one of the 23 chromosomes (except for the acrocentric chromosomes). The p-arm and the q-arm probes for each chromosome are labeled in different colors with two spectrally independent fluorophores (SpectrumGreen and SpectrumOrange).

All digital images were captured using a Leitz microscope (Leica DM RA 2 or Leica DM 5000B, Leica Microsystems GmbH, Leica Microsystems, Milan, Italy) equipped with a charge coupled device (CCD) camera (Leica Microsystems) and analyzed by means of various software (Leica CW4000 or Chromowin). All images were captured at 100× magnification. Some of them were then enlarged photographically.

### 4.3. Array Comparative Genomic Hybridization

Array Comparative Genomic Hybridization (Array CGH) analysis, was performed using SurePrint G3 Human Microarray kit 2x400 K (for 7qs case) and SurePrint G3 Human CGHplusSNP Microarray 4x180 K (for 17ps case) following the manufacturer's instructions (Agilent Technologies, Palo Alto, CA, USA). Genomic DNA from peripheral blood samples from patients and their parents was extracted using Wizard Genomic DNA Purification Kit (PromegaTM, Mannheim, Germany) according to the manufacturer's instructions. DNA concentration was determined on a NanoDrop ND-1000 spectrophotometer (NanoDrop Technologies, Berlin, Germany).

The array was scanned at 2-μm/3-μm resolution using Agilent microarray scanner and analyzed using Feature Extraction v10.7 and Genomic Workbench v7.0 (Agilent Technologies) in order to read scanner image and to calculate copy number variations. Significant chromosomal aberration was determined using the algorithm ADM-2 (threshold, 5.0; absolute minimum average log2 ratio, 0.20; with at least three or more consecutive probe sets; see more detailed in [35]).

### 4.4. UCSC Genome Browser

Segmental duplications reported in Figures 7 and 8 were collected from the UCSC genome browser (https://genome.ucsc.edu/cgi-bin/hgGateway) selecting only the telomeric regions of non-acrocentric chromosomes and p-arms of acrocentric chromosomes. Segmental duplications track can be found in the Repeats group.

## 5. Conclusions

Finally, we stress that our results provide evidence supporting the role of cytogenetics as an omics approach also in the era of whole genome sequencing.

**Supplementary Materials:** The following are available online at www.mdpi.com/xxx/s1, Figure S1. Examples of acrocentric chromosomes association in QFQ banded chromosome preparations.

**Author Contributions:** Conceptualization, S.R., L.D., A.B.; methodology, S.R., L.D., A.B.; formal analysis, S.R., L.D., A.B.; investigation, S.R., L.D., A.B.; resources, N.V., E.S., F.C., C.C., I.C., M.G., L.R., E.M., A.P., R.M., M.P.R.; data curation, S.R., N.V., E.S., F.C.; writing—original draft preparation, S.R., D.C., L.D., A.B.; writing—review and editing, S.R., D.C., N.V., E.S., F.C., L.R., P.R., L.D., M.L., G.R., A.B.; visualization, S.R., D.C., L.D., A.B.; supervision, S.R., L.D., P.R., A.B.; project administration, L.D., A.B.; funding acquisition, M.L. and A.B. All authors have read and agreed to the published version of the manuscript.

**Acknowledgments:** The authors would like to express their very great appreciation to Chiara Cilibrasi for her valuable support in the manuscript revision.

**Funding:** This research was funded by 2017-NAZ-0247, 2018-ATE-0406 grants from University of Milano-Bicocca (to Angela Bentivegna) and by ADOPTBBMRI-ERIC:implementAtion anD OPeration of the gaTeway for health into the Biobanking and BioMolecular resources Research Infrastructure -European Research Infrastructure Consortium from Horizon 2020 (to Marialuisa Lavitrano) Call:

H2020-INFRADEV-1-2015-1 Topic: INFRADEV-3-2015 Type of action: RIA Proposal number: 676550 Proposal acronym: ADOPT BBMRI-ERIC.

**Conflicts of Interest:** The authors declare no conflict of interest.

Abbreviations

| | |
|---|---|
| CGH | Comparative genomic hybridization |
| FISH | Fluorescent in situ hybridization |
| ISCN | International System for Cytogenomic Nomenclature |
| NAHR | Non- allelic homologous recombination |
| PHA | Phytohemagglutinin |
| rDNA | Ribosomal DNA |

## References


1. Klein, S.J.; O'Neill, R.J. Transposable elements: Genome innovation, chromosome diversity, and centromere conflict. *Chromosome Res.* **2018**, *26*, 5–23.
2. Dumbovic, G.; Forcales, S.V.; Perucho, M. Emerging roles of macrosatellite repeats in genome organization and disease development. *Epigenetics* **2017**, *12*, 515–526.
3. de Koning, A.P.J.; Gu, W.; Castoe, T.A.; Batzer, M.A.; Pollock, D.D. Repetitive elements may comprise over two-thirds of the human genome. *PLoS Genet.* **2011**, *7*, e1002384.
4. Sylvester, J.E.; Whiteman, D.A.; Podolsky, R.; Pozsgay, J.M.; Respess, J.; Schmickel, R.D. The human ribosomal RNA genes: Structure and organization of the complete repeating unit. *Hum Genet.* **1986**, *73*, 193–198.
5. McStay, B. Nucleolar organizer regions: Genomic 'dark matter' requiring illumination. *Genes Dev.* **2016**, *30*, 1598–1610.
6. Floutsakou, I.; Agrawal, S.; Nguyen, T.T.; Seoighe, C.; Ganley, A.R.; McStay, B. The shared genomic architecture of human nucleolar organizer regions. *Genome Res*. **2013**, *23*, 2003–2012.
7. Hamerton, J.; Canning, N.; Ray, M.; Smith, J. A cytogenetic survey of 14,069 newborn infants. I. Incidence of chromosomal abnormalities. *Clin. Genet.* **1975**, *8*, 223–243.
8. Louzada, S.; Lopes, M.; Ferreira, D.; Adega, F.; Escudeiro, A.; Gama-Carvalho, M.; Chaves, R. Decoding the Role of Satellite DNA in Genome Architecture and Plasticity—An Evolutionary and Clinical Affair. *Genes* **2020**, *11*, 72.
9. Cazaux, B.; Catalan, J.; Veyrunes, F.; Douzery, E.J.; Britton-Davidian, J. Are ribosomal DNA clusters rearrangement hotspots? A case study in the genus Mus (Rodentia, Muridae). *BMC Evol. Biol.* **2011**, *11*, 124.
10. Richard, G.F.; Kerrest, A.; Dujon, B. Comparative genomics and molecular dynamics of DNA repeats in eukaryotes. *Microbiol. Mol. Biol. Rev.* **2008**, *72*, 686–727.
11. Cockwell, A.E.; Jacobs, P.A.; Crolla, J.A. Distribution of the D15Z1 copy number polymorphism. *Eur. J. Hum. Genet.* **2007**, *15*, 441–445.
12. Ramos, S.; Rodríguez, R.; Castro, O.; Grether, P.; Molina, B.; Frias, S. Presence of 15p Marker D15Z1 on the Short Arm of Acrocentric Chromosomes is Associated with Aneuploid Offspring in Mexican Couples. *Int. J. Mol. Sci.* **2019**, *20*, 5251.
13. International Standing Committee on Human Cytogenetic Nomenclature. *ISCN: An International System for Human Cytogenetic Nomenclature*; Karger: Basel, Switzerland, 1995.
14. Sarri, C.; Douzgou, S.; Gyftodimou, Y.; Tümer, Z.; Ravn, K.; Pasparaki, A.; Sarafidou, T.; Kontos, H.; Kokotas, H.; Karadima, G.; et al. Complex distal 10q rearrangement in a girl with mild intellectual disability: Follow up of the patient and review of the literature of non-acrocentric satellited chromosomes. *Am. J. Med. Genet. A* **2011**, *155A*, 2841–2854.
15. Feldman, G.L.; Weiss, L.; Phelan, M.C.; Schroer, R.J.; Van Dyke, D.L. Inverted duplication of 8p: Ten new patients and review of the literature. *Am. J. Med. Genet.* **1993**, *47*, 482–486.
16. Guo, W.J.; Callif-Daley, F.; Zapata, M.C.; Miller, M.E. Clinical and cytogenetic findings in seven cases of inverted duplication of 8p with evidence of a telomeric deletion using fluorescence in situ hybridization. *Am. J. Med. Genet*. **1995**, *58*, 230–236.
17. Floridia, G.; Piantanida, M.; Minelli, A.; Dellavecchia, C.; Bonaglia, C.; Rossi, E.; Gimelli, G.; Croci, G.; Franchi, F.; Gilgenkrantz, S.; et al. The same molecular mechanism at the maternal meiosis I produces mono and dicentric 8p duplications. *Am. J. Hum. Genet.* **1996**, *58*, 785–796.

18. Reeves, R.; Nissen, M.S. The A.T-DNA-binding domain of mammalian high mobility group I chromosomal proteins. A novel peptide motif for recognizing DNA structure. *J. Biol. Chem*. **1990**, *265*, 8573–8582.
19. Gu, W.; Zhang, F.; Lupski, J.R. Mechanisms for human genomic rearrangements. *Pathogenetics* **2008**, *1*, 4.
20. Zuffardi, O.; Bonaglia, M.; Ciccone, R.; Giorda, R. Inverted duplications deletions: Underdiagnosed rearrangements?? *Clin. Genet*. **2009**, *75*, 505–513.
21. van Koningsbruggen, S.; Gierlinski, M.; Schofield, P.; Martin, D.; Barton, G.J.; Ariyurek, Y.; den Dunnen, J.T.; Lamond, A.I. High-resolution whole-genome sequencing reveals that specific chromatin domains from most human chromosomes associate with nucleoli. *Mol. Biol. Cell*. **2010**, *21*, 3735–3748.
22. Conconi, D.; Villa, N.; Redaelli, S.; Sala, E.; Crosti, F.; Maitz, S.; Rigoldi, M.; Parini, R.; Dalprà, L.; Lavitrano, M.; et al. Familiar unbalanced complex rearrangements involving 13 p-arm: Description of two cases. *Mol. Cytogenet*. **2018**, *11*, 52.
23. Villa, N.; Sala, E.; Colombo, D.; Dell'Orto, M.; Dalprà, L. Monosomy and trisomy 1q44-qter in two sisters originating from a half cryptic 1q;15p translocation. *J. Med. Genet.* **2000**, *37*, 612–615.
24. Villa, N.; Scatigno, A.; Redaelli, S.; Conconi, D.; Cianci, P.; Farina, C.; Fossati, C.; Dalprà, L.; Maitz, S.; Selicorni, A. 14q32.3-qter trisomic segment: A case report and literature review. *Mol. Cytogenet*. **2016**, *9*, 60.
25. Ramírez, M.J.; Surrallés, J. Laser confocal microscopy analysis of human interphase nuclei by three-dimensional FISH reveals dynamic perinucleolar clustering of telomeres. *Cytogenet. Genome Res.* **2008**, *122*, 237–242.
26. Stimpson, K.M.; Sullivan, L.L.; Kuo, M.E.; Sullivan, B.A. Nucleolar organization, ribosomal DNA array stability, and acrocentric chromosome integrity are linked to telomere function. *PLoS ONE* 2014, *9*, e92432.
27. Eichler, E.E.; Hoffman, S.M.; Adamson, A.A.; Gordon, L.A.; McCready, P.; Lamerdin, J.E.; Mohrenweiser, H.W. Complex beta-satellite repeat structures and the expansion of the zinc finger gene cluster in 19p12. *Genome Res.* **1998**, *8*, 791–808.
28. Hirai, H.; Taguchi, T.; Godwin, A.K. Genomic differentiation of 18S ribosomal DNA and beta-satellite DNA in the hominoid and its evolutionary aspects. *Chromosome Res.* **1999**, *7*, 531–540.
29. Cardone, M.F.; Ballarati, L.; Ventura, M.; Rocchi, M.; Marozzi, A.; Ginelli, E.; Meneveri, R. Evolution of beta satellite DNA sequences: Evidence for duplication-mediated repeat amplification and spreading. *Mol. Biol. Evol.* **2004**, *21*, 1792–1799.
30. Bersaglieri, C.; Santoro, R. Genome Organization in and around the Nucleolus. *Cells* **2019**, *8*, 579.
31. Nakagawa, T.; Okita, A.K. Transcriptional silencing of centromere repeats by heterochromatin safeguards chromosome integrity. *Curr. Genet.* **2019**, *65*, 1089–1098.
32. Diagnostica Citogenetica, Consensus 1995. Associazione Italiana di Citogenetica Medica. *Analysis* **1995**, *8, 12–42*.
33. Verma, R.S.; Babu, A. *Human Chromosomes: Manual of Basic Techniques*; McGraw-Hill: New York, USA, 1995.
34. International Standing Committee on Human Cytogenomic Nomenclature. *ISCN: An International System for Human Cytogenomic Nomenclature*; Karger: Basel, Switzerland, 2016.
35. Redaelli, S.; Maitz, S.; Crosti, F.; Sala, E.; Villa, N.; Spaccini, L.; Selicorni, A.; Rigoldi, M.; Conconi, D.; Dalprà, L.; et al. Refining the Phenotype of Recurrent Rearrangements of Chromosome 16. *Int. J. Mol. Sci.* **2019**, *20*, 1905.

*© 2020 by the authors. Licensee MDPI, Basel, Switzerland. This article is an open access article distributed under the terms and conditions of the Creative Commons Attribution (CC BY) license (http://creativecommons.org/licenses/by/4.0/).*
